# Highly Active Ni- and Co-Based Bimetallic Catalysts for Hydrogen Production From Ammonia-Borane

**DOI:** 10.3389/fchem.2019.00138

**Published:** 2019-03-20

**Authors:** Shinya Furukawa, Genki Nishimura, Tomoaki Takayama, Takayuki Komatsu

**Affiliations:** ^1^Institute for Catalysis, Hokkaido University, Sapporo, Japan; ^2^Elements Strategy Initiative for Catalysts and Batteries, Kyoto University, Kyoto, Japan; ^3^Department of Chemistry, School of Science, Tokyo Institute of Technology, Tokyo, Japan

**Keywords:** hydrogen production, ammonia borane, hydrolysis, alloy, catalyst

## Abstract

Ammonia-borane is one of the most promising candidates for hydrogen carriers. A series of Ni- and Co-based bimetallic catalysts supported on SiO_2_ (Ni–M/SiO_2_ and Co–M/SiO_2_; M = Ga, Ge, Sn, Zn) was prepared and tested as catalysts for hydrogen production from ammonia-borane (AB) in water or methanol. Ni–Zn/SiO_2_ and Co–Ge/SiO_2_ exhibited catalytic activities much higher than those of monometallic Ni/SiO_2_ and Co/SiO_2_, respectively. Ni–Zn/SiO_2_ showed a high catalytic activity when water was used as a solvent, where the reaction was completed within 6 min at room temperature with a specific reaction rate of 4.3 ml min^−1^ mmol-cat^−1^ mM-AB^−1^. To the best of our knowledge, this is the highest value among those reported using 3d metal-based catalysts. Co–Ge/SiO_2_ afforded a five-fold higher reaction rate than that of the corresponding monometallic Co/SiO_2_. XRD, TEM, and HAADF-STEM-EDS analyses revealed that Ni_0.75_Zn_0.25_ and Co_0.8_Ge_0.2_ solid-solution alloys were formed with high phase purities. An XPS study showed that Co atoms in Co_0.8_Ge_0.2_ were electron-enriched due to electron transfer from Ge to Co, which may be the origin of the improved catalytic activity.

## Introduction

Hydrogen has been considered as one of the best energy carrier alternatives to fossil fuels because of its high energy density, clean combustion product (only water), and environmental friendliness (Schlapbach and Züttel, 2011). Hydrogen is currently produced by steam reforming of methane contained in natural gas (Heinzel et al., 2002), which is not renewable and sustainable. Therefore, alternative methodologies such as photocatalytic (Moniz et al., 2015; Chen et al., 2017; Wang et al., 2017) and photoelectrochemical (Zhang et al., 2014; Zheng and Zhang, 2016; Han et al., 2017) water splitting have recently attracted increasing attention. On the other hand, storage, transport, and release of hydrogen are known as technological barriers to practical application in view of cost and safety (Züttel, 2003). Ammonia–borane (H_3_N·BH_3_, hereafter AB) is one of the more promising candidates as a hydrogen carrier or source (Landge et al., 2018) owing to its high hydrogen content (19.6 wt%), high thermal stability, and low toxicity (Marder, 2007). AB can release three equimolar amounts of hydrogen at ambient temperatures using an appropriate catalyst through solvolysis with protic solvents such as water and methanol. Using noble metals such as Pt, Rh, Ir enables the achievement of rapid hydrogen release typically within several minutes (Chandra and Xu, 2007; Xu and Chandra, 2007). Recently, development of non-noble 3d transition metal-based catalysts that are active for hydrogen production from AB has been increasingly focused (Xu and Chandra, 2006; Yan et al., 2008, 2009; Kalidindi et al., 2009; Metin et al., 2010; Patel et al., 2010; Ozay et al., 2011; Peng et al., 2015). To develop highly efficient catalytic systems using base metal elements, the catalytic activity of these metals should be greatly enhanced by appropriate catalyst design such as the modification of morphology, the addition of some cocatalysts, or the formation of alloy phases (Furukawa and Komatsu, 2017).

In this study, a series of Ni- and Co-based bimetallic catalysts was prepared (Ni–M/SiO_2_ and Co–M/SiO_2_; M = Ga, Ge, Sn, and Zn) and tested as catalysts for hydrogen production from AB in water or methanol as a solvent. The observed catalytic performances were discussed in view of the reaction mechanism. Herein, we report a novel and highly efficient catalytic system for hydrogen production from AB using non-noble metal catalysts.

## Materials and Methods

### Catalyst Preparation

Monometallic Ni and Co catalysts were prepared by pore-filling impregnation using silica as a support. Aqueous solutions of Ni(NO_3_)_2_·6H_2_O (Wako, 99%) or Co(NO_3_)_2_·3H_2_O (Sigma Aldrich, 99%) were added to dried silica gel (CARiACT G-6, Fuji Silysia, *S*_BET_ = 470 m^2^ g^−1^) so that the solutions filled the silica pores. The mixtures were sealed overnight at room temperature and dried over a hot plate, followed by reduction under flowing H_2_ at 600°C for 1 h. Silica-supported Ni- and Co-based catalysts (Ni–M/SiO_2_ and Co–M/SiO_2_; M = Ga, Ge, Sn, and Zn) were prepared by pore-filling co-impregnation. Mixed aqueous solutions of Ni(NO_3_)_2_·6H_2_O or Co(NO_3_)_2_·3H_2_O and a second metal salt, Ga(NO_3_)_3_·nH_2_O (Wako, 99.9%), (NH_4_)_2_GeF_6_ (Sigma Aldrich, 99.99%), SnCl_2_ (Kanto, 97%), and Zn(NO_3_)_2_·6H_2_O (Kanto, 99%) were used in a manner similar to that of the monometallic catalyst. The metal loading of Ni or Co, the atomic ratio of Ni/M or Co/M, and the reduction temperature during the catalyst preparation were set to 3 wt%, 3.0, and 600°C except for Ni–Ge and Co–Ge, which had a reduction temperature of 800°C, and Co–Sn with an atomic ratio of 1.0.

### Characterizations

The crystal structures of the catalysts were determined by powder X-ray diffraction (XRD) with a Rigaku RINT2400 diffractometer using a Cu Kα X-ray source. Difference XRD patterns were obtained by subtracting the pattern for the SiO_2_ support from those of the supported catalysts. Transmission electron microscopy (TEM) was conducted using a JEOL JEM-2010F microscope at an accelerating voltage of 200 kV. To prepare the TEM specimen, all samples were sonicated in tetrachloromethane and then dispersed on a Cu grid supported by an ultrathin carbon film. X-ray photoelectron spectra (XPS) of the bimetallic compounds were measured with an ULVAC PHI 5000 VersaProbe spectrometer. The catalyst was pressed into a pellet and placed into a quartz reactor, where it was reduced under flowing hydrogen (60 ml min^−1^) at 450°C for 0.5 h prior to the measurement. The sample was put into a transfer vessel in a grove box (O_2_ concentration: < 1 ppm) and then introduced into the spectrometer without exposure to air. Spectra were obtained with an Al Kα X-ray source, using C 1s as a reference for binding energy (284.8 eV). The reduction behavior of the catalyst was examined by temperature-programmed reduction (TPR). Under flowing H_2_ (5%)/Ar, the temperature of the sample bed was raised from room temperature to 900°C at a heating rate of 10°C·min^−1^ and the consumption of hydrogen was continuously measured by a thermal conductivity detector (TCD).

### Catalytic Reaction

A catalyst (100 mg) was placed into a 50 mL three-necked round-bottom flask equipped with a silicone rubber septum and a gas burette and pretreated under an H_2_ stream (60 mL·min^−1^) at 450°C for 0.5 h using a mantle heater. After the pretreatment, dry Ar (20 mL·min^−1^) was passed into the flask to replace the residual H_2_, and the flask was cooled to room temperature. A reaction mixture containing a solvent (deionized water or dehydrated methanol, Kanto 99.8%, 10 mL) and AB (Sigma-Aldrich, 97%, 2.0 mmol) was added into the flask through the septum at 25°C. The volume of the evolved H_2_ was measured using the gas burette. The total volume of H_2_ is expected to be 147 ml (6.0 mmol) at 25°C for the complete conversion of AB (2.0 mmol).

## Results and Discussion

**Figure 1:**
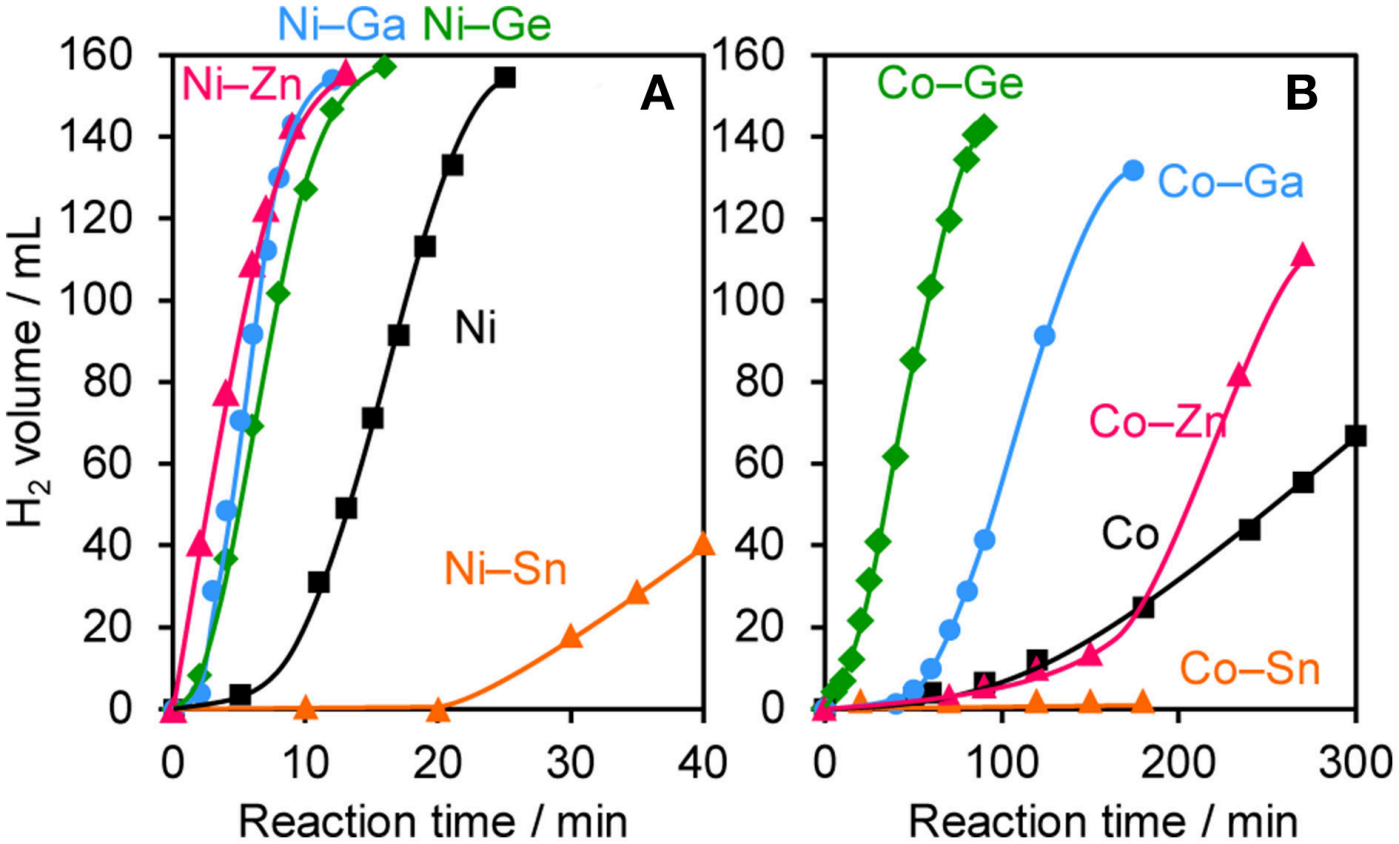
Time course of H_2_ evolution during catalytic methanolysis of AB using **(A)** Ni- and **(B)** Co-based catalysts.

**Figure 2:**
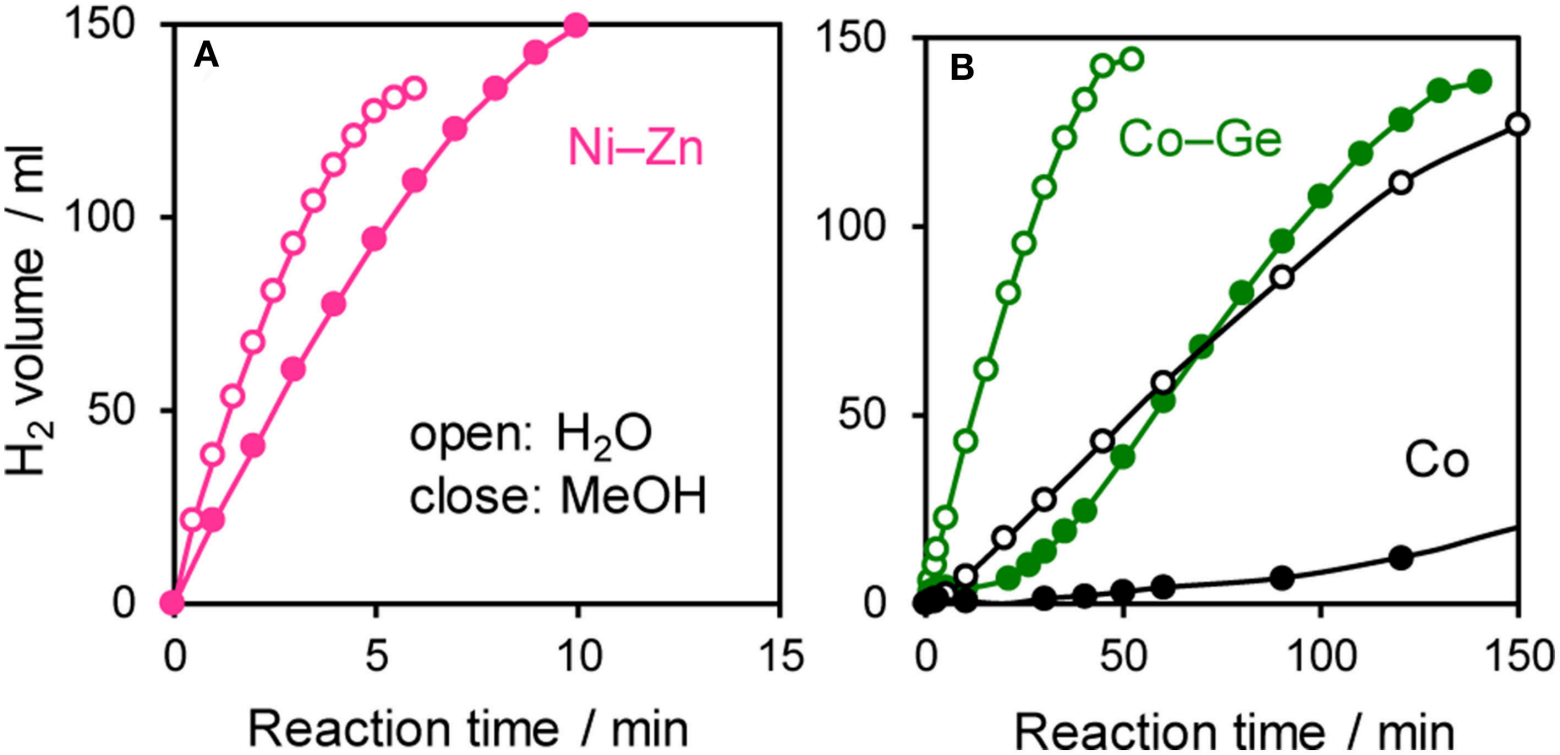
Time course of H_2_ evolution during catalytic hydrolysis (H_2_O) and methanolysis (MeOH) of AB using **(A)** Ni–Zn, **(B)** Co–Ge, and Co catalyst.

**Figure 3:**
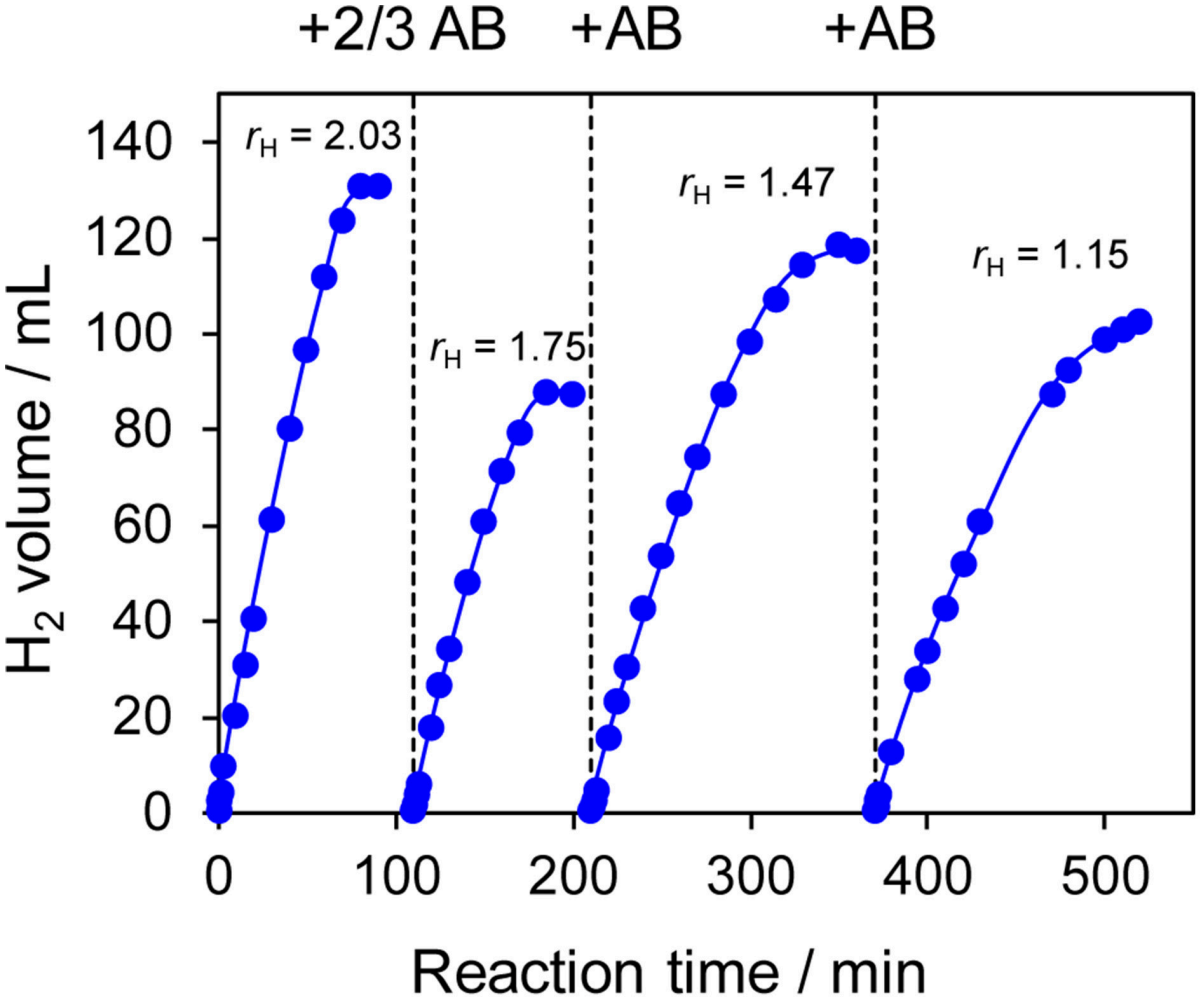
Reuse tests for H_2_ production in methanol using Co–Ge/SiO_2_ catalyst. For the first reuse at 110 min, the amount of AB was reduced to 2/3 (1.33 mmol).

The prepared Ni- and Co-based catalysts were tested for H_2_ production from AB using methanol as a solvent (Figure 1). The monometallic Ni catalyst showed moderate catalytic activity with a short induction period. The Ni–Sn catalyst showed a very low catalytic activity with a long induction period. On the other hand, the Ni–Zn, Ni–Ga, and Ni–Ge catalysts exhibited high catalytic activities without an induction period. Particularly for the Ni–Zn catalyst, the reaction was completed within ca. 10 min. Co-based catalysts generally gave catalytic activities lower than those of Ni-based catalysts, which reflects the intrinsic difference of catalytic activity between Ni and Co for this reaction. Moreover, the catalytic activities were quite different depending on the second metal (Co–Ge > Co–Ga >> Co >> Co–Sn). Induction periods were observed for some catalysts (Ni, Ni–Sn, Co, Co–Ga, and Co–Zn), indicating that, for these catalysts, some real active species (probably, zero-valent Ni or Co species) were formed or increased during the catalytic reaction. A remarkable enhancement in the reaction rate was achieved by addition of Ge to Co, suggesting the contribution of a specific effect of Ge on the catalysis. The discovered active catalysts, Ni–Zn and Co–Ge, were also tested in hydrogen production from AB in water as a solvent (Figure 2). For each catalyst, H_2_ evolution in water was much faster than that in methanol as reported for Ni- and Co-based catalysts in the literature. Note that the reaction was completed within 6 min when the Ni–Zn catalyst was used. This is one of the best performances in AB hydrolysis reported to this day. The obtained catalytic performance was listed in Table 1 with those of reported catalysts. Because the reaction conditions were different depending on each study, we quantitatively compared them based on the specific H_2_ formation rates (*r*_cat[AB]_) calculated by dividing H_2_ formation rates (*r*_H2_) by the amount of catalyst (mmol) and concentration of AB (mM). Since the number of active sites, such as metal atoms on the surface, was not clearly mentioned, the total number of metal atoms was used for this calculation. The difference in AB concentration was compensated for by assuming the first-order dependence of the formation rate on AB concentration. The Ni–Zn catalyst exhibited an *r*_cat[AB]_ value higher than that of Ni_2_P, which was reported to be the most active base-metal catalyst for AB hydrolysis (Peng et al., 2015). Therefore, the Ni–Zn catalyst is the most active 3d metal-based catalyst for H_2_ production by hydrolysis of AB. We also examined the reusability of the catalysts. Figure 3 shows the result of the reuse test for the Co–Ge/SiO_2_ catalyst in H_2_ production in methanol. At the first reuse at 110 min, we added 1.33 mol of AB (2/3 equivalent to the standard condition). The total volume H_2_ evolved decreased from 130.5 to 87 ml, showing complete conversion of AB at reuse. The reaction rate also became approximately two-thirds of the original (2.03–1.75), indicating that *r*_H2_ strongly depends on AB concentration. Although the reaction rate was slightly decreased at the second and third reuse, the Co–Ge/SiO_2_ catalyst could be reused without any regeneration procedure. Thus, it was found that the Co–Ge/SiO_2_ was a recyclable heterogeneous catalyst for H_2_ production from AB.

**Table 1:** Summary of reaction condition and rate of AB hydrolysis at 25°C using various catalysts.

**Catalyst**	**Cat (mmol)**	**AB (mmol)**	**AB/Cat**	***r*_**H2**_ (ml min^**−1**^)**	***r*_**cat**_ (ml min^**−1**^ mmol-cat^**−1**^)**	***r*_**cat[AB]**_ (ml min^**−1**^ mmol-cat^**−1**^ mM-AB^**−1**^)**	**Duration (min)^**a**^**	**References**
Fe	0.192	1.6	8.3	15	79	0.50	10	Yan et al., 2008
Co/C	0.058	3.2	56	3.6	61	0.20	50	Xu and Chandra, 2006
Co–B	0.2	15.0	75	29	145	1.45	–	Patel et al., 2010
Ni	0.025	2.0	80	5.0	200	1.00	50	Metin et al., 2010
Ni_2_P	0.054	2.08	38	46	852	2.05	6	Peng et al., 2015
Ni–Zn	0.050	2.0	40	43	860	4.30	6	This work
Co–Ge	0.051	2.0	39	4.5	88	0.44	50	
Co	0.051	2.0	39	0.9	18	0.09	>150	

a *Time needed for completion of H_2_ evolution*.

**Figure 4:**
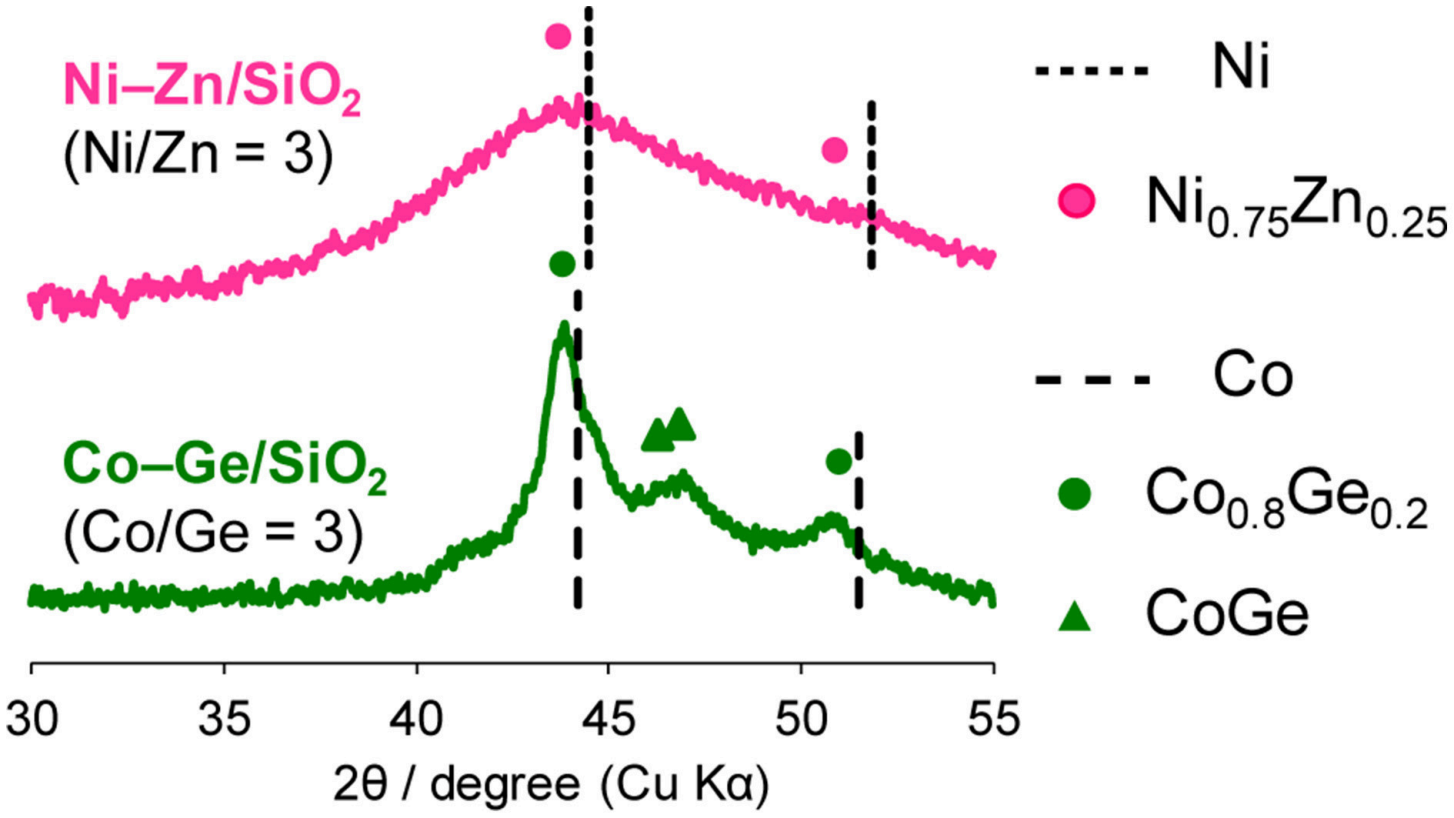
XRD patterns of Ni–Zn/SiO_2_ (Ni/Zn = 3) and Co–Ge/SiO_2_ (Co/Ge = 3).

**Figure 5:**
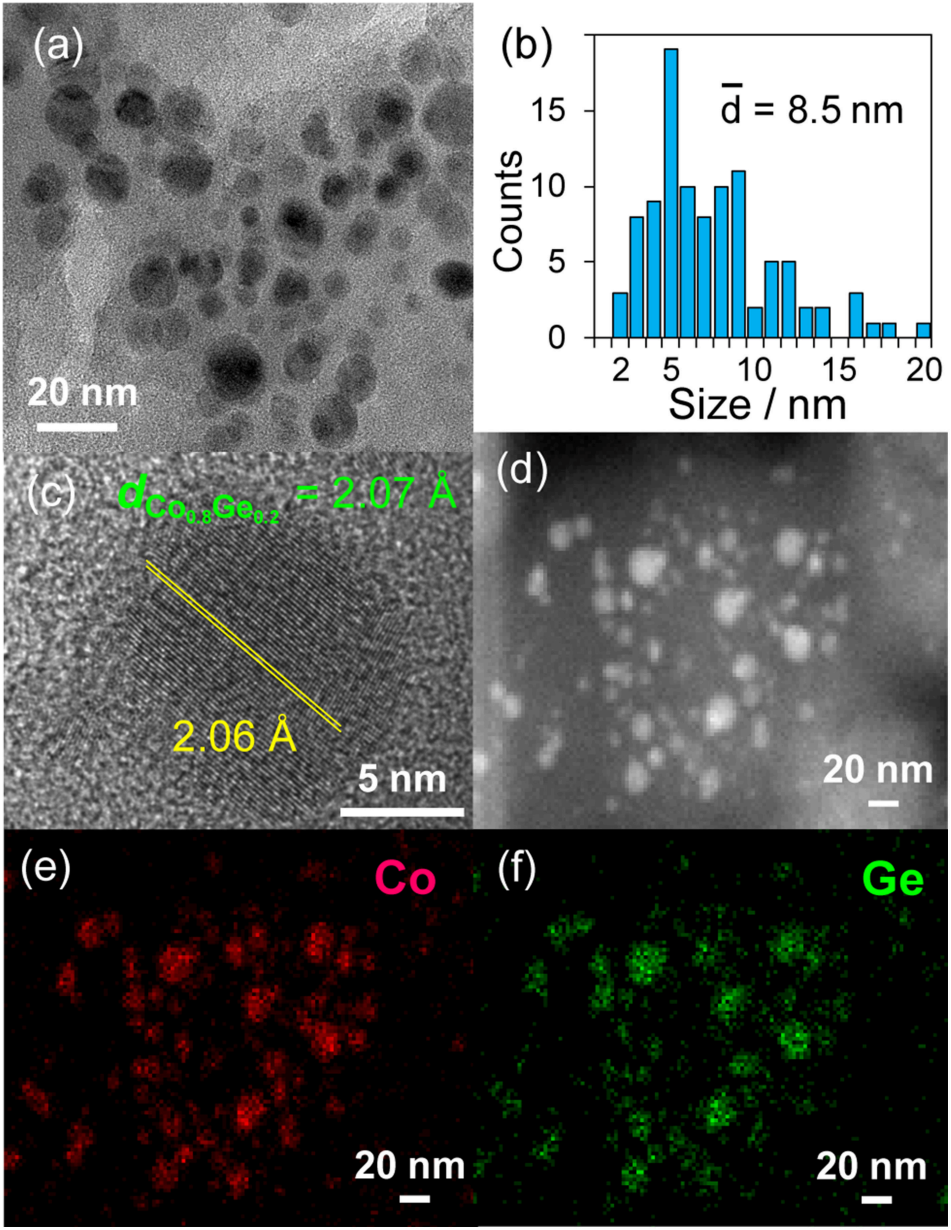
**(a)** TEM image and the **(b)** size distribution of Co–Ge/SiO_2_ catalyst. **(c)** High resolution TEM image of a single nanoparticle of Co–Ge. **(d)** HAAF-STEM image of Co–Ge/SiO_2_ catalyst and elemental maps of **(e)** Co, and **(f)** Ge for the nanoparticles acquired using EDS.

Then, the discovered active catalysts were characterized to clarify their structures. Figure 4 shows XRD patterns of the Ni–Zn/SiO_2_ and Co–Ge/SiO_2_ catalysts. For Ni–Zn/SiO_2_, a solid-solution alloy between Ni and Zn with a 3: 1 ratio, namely Ni_0.75_Zn_0.25_ phase (Vassilev, 1992), was observed as single phase. A similar alloy phase (Co_0.8_Ge_0.2_) (Ishida and Nishizawa, 1991) was also observed as a main species for Co–Ge/SiO_2_. One-to-one intermetallic phase of CoGe was also detected as a minor species. Thus, XRD analysis confirmed the formation of alloy phases with high purities. The crystallite sizes of Ni–Zn and Co–Ge were estimated using Scherrer equation as <3 and 9 nm, respectively. The larger crystallite size of Co–Ge may stem from the higher reduction temperature (800°C) during the catalyst preparation. Figure 5 shows TEM and STEM images of Co–Ge/SiO_2_, size distribution of nanoparticles, and the elemental map of Co and Ge acquired using EDS. Particle size ranged from 2 to 20 nm with a mean diameter of 8.5 nm (Figures 5a,b), which is consistent with the crystallite size estimated by the Scherrer equation (9 nm). Figure 5c displays the high-resolution TEM image of a single Co–Ge nanoparticle. Lattice fringes with 2.06 spacing were clearly observed, which agrees finely with the interplanar distance of the (111) plane of Co_0.8_Ge_0.2_ solid-solution alloy with an fcc structure (2.07) (Ishida and Nishizawa, 1991). The elemental maps of Co and Ge that were acquired using the EDS analysis revealed that the Co and Ge atoms comprising the nanoparticles were homogeneously dispersed (Figures 5d–f). These results strongly suggest that the Co_0.8_Ge_0.2_ alloy nanoparticles were formed with high phase purities. Thus, the results obtained from the STEM-EDS analysis were consistent with that of XRD.

We then studied the reason why the catalytic activity was significantly enhanced by the formation of the alloy phase. Because the Co–Ge system exhibited a remarkable increase in the reaction rate (five times higher) compared to pure Co, we focused on the difference between the Co and Co–Ge systems.

**Figure 6:**
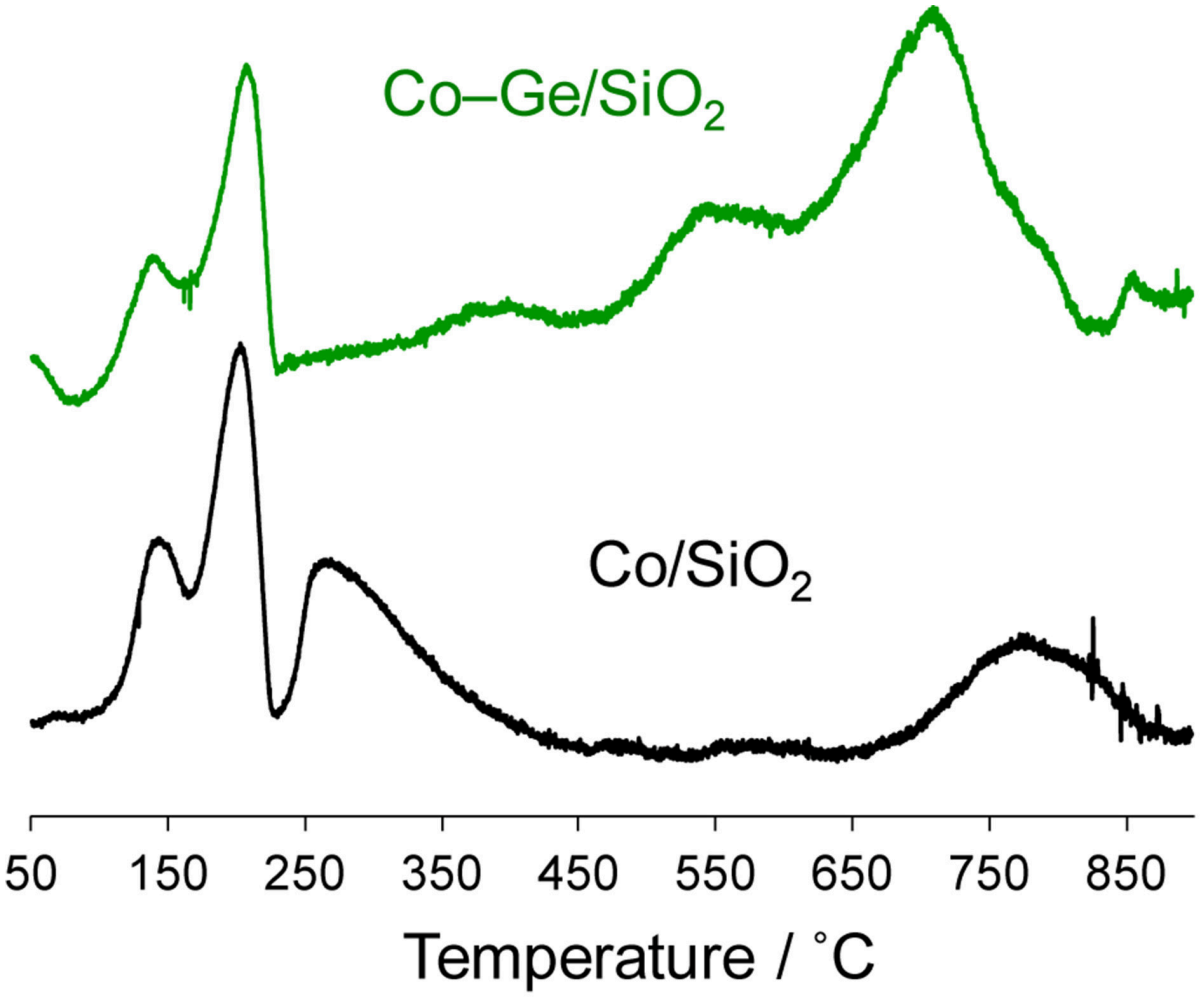
TPR profiles for as-impregnated Co(NO_3_)_2_/SiO_2_ and Co(NO_3_)_2_-(NH_4_)_2_GeF_6_/SiO_2_ catalysts.

Figure 6 shows the TPR profiles of the Co/SiO_2_ and Co–Ge/SiO_2_ catalysts. To evaluate the reduction temperatures of the component metals, as-impregnated catalysts were used for the TPR experiments. For both catalysts, intense peaks were observed at 150 and 200°C, which may be attributed to the reduction of O_2_ or NO_2_ derived from the decomposition of the Co(NO_3_)_2_ (van Steen et al., 1996). For Co/SiO_2_, two reduction peaks were observed at 260 and 760°C, which are assigned to the reduction of Co^2+^ species that weakly and strongly interact with the silica surface, respectively (van Steen et al., 1996). On the contrary, for Co–Ge/SiO_2_, three different reduction peaks were observed at 370, 550, and 740°C, which could be assigned to the reduction of Co^2+^, Co^3+^, and Ge^4+^ species. We previously reported a TPR profile of Ge/SiO_2_, with reduction peaks appearing at temperatures higher than 650°C (Komatsu et al., 1997). Therefore, the reduction peak at 740°C can be attributed to the reduction of Ge^4+^. The other reduction peaks assignable for Co species appeared at much higher temperatures than that for Co/SiO_2_, suggesting that the strong interaction between Co and Ge inhibits the reduction of Co^2+^. It can be said that Co species in Co/SiO_2_ were not completely reduced because the reduction temperature for the preparation of Co/SiO_2_ (600°C) is not sufficient to completely reduce all the Co species. This may be one of the reasons why the Co/SiO_2_ catalysts showed low catalytic activity. On the contrary, for Co–Ge/SiO_2_, the reduction temperature for the preparation (800°C) is enough to reduce all the Co^2+^ and Ge^4+^ species.

**Figure 7:**
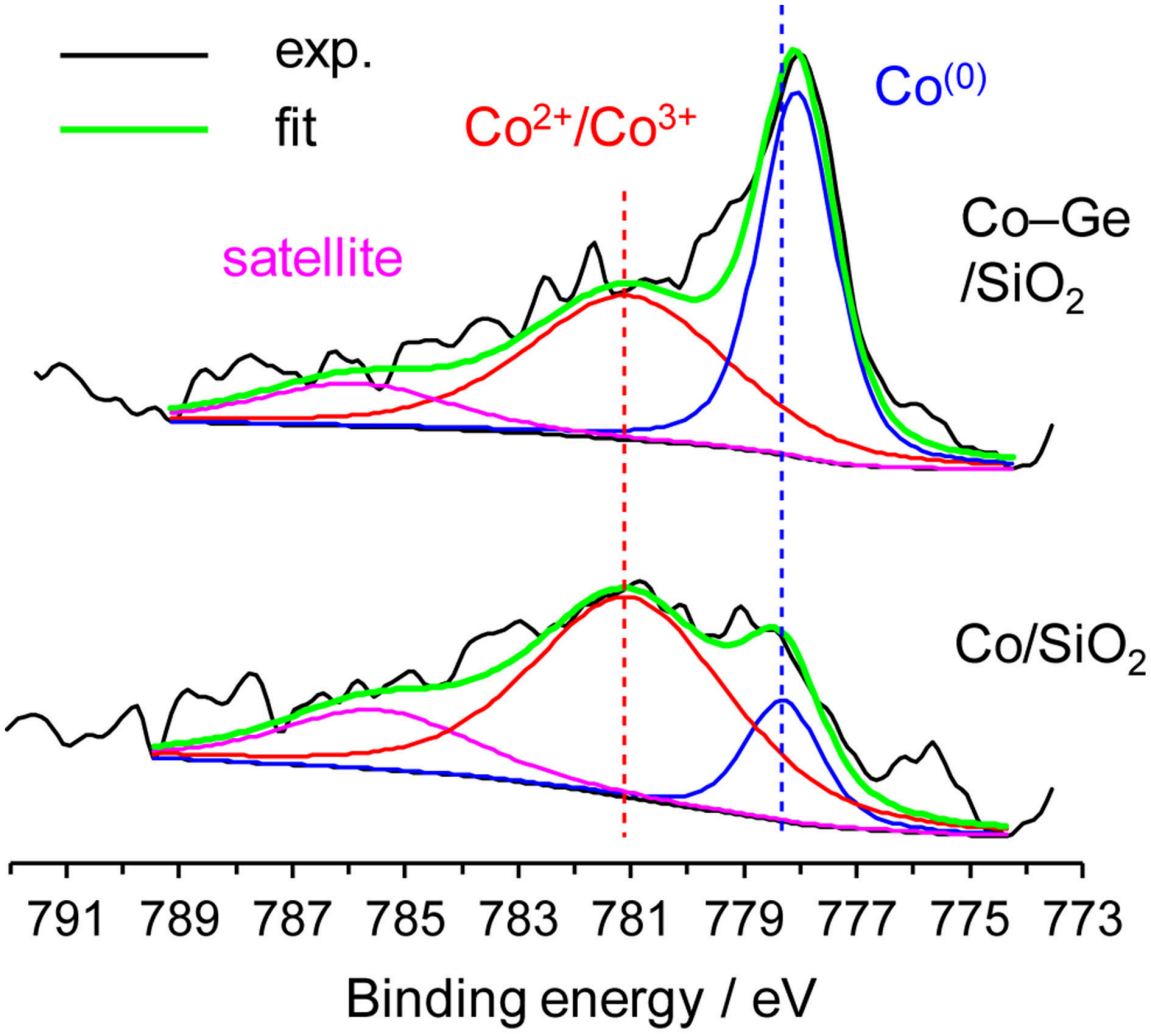
Co 2p_3/2_ XPS of Co/SiO_2_ and Co–Ge/SiO_2_ catalysts.

We also investigated the electronic states of the Co/SiO_2_ and Co–Ge/SiO_2_ catalysts by XPS (Figure 7). For Co/SiO_2_, the fraction of Co^2+^ or Co^3+^ species (Sexton et al., 1986) was much higher than that of Co^(0)^, suggesting that a large part of the catalyst surface is oxidized. By contrast, for Co–Ge/SiO_2_, Co^(0)^ (Sexton et al., 1986) was detected as a main species. These results are consistent with the expectation derived from TPR mentioned above. It is also likely that Co atoms on the Co–Ge/SiO_2_ catalyst are more resistant to aerobic oxidation than those on Co/SiO_2_. Jagirdar et al. reported that Co^2+^ or Ni^2+^ ions could be reduced by the evolved H_2_ during hydrolysis of AB to form Ni or Co nanoparticles (Kalidindi et al., 2008). Therefore, it is likely that similar *in situ* reduction of Co^2+^ could occur to completely reduce the catalyst surface in our system. This may be the reason why the induction period was observed in H_2_ production from AB over Co/SiO_2_. The binding energies of Co^2+^ or Co^3+^ for Co/SiO_2_ and Co–Ge/SiO_2_ were almost same, suggesting that Ge species do not affect the electronic state of Co cations. However, a different trend was observed for Co^(0)^ species: Co–Ge/SiO_2_ exhibited a lower binding energy than Co/SiO_2_, indicating that Co atoms in Co–Ge are electron-enriched compared with pure Co. This is probably because electron transfer from Ge to Co-occurs due to alloy formation. Similar electron transfer has also been reported for the relevant systems such as Pt–Ge alloys (Komatsu et al., 1997). Thus, XPS analysis revealed that the formation of Co–Ge alloy drastically changed the electronic state of Co. On the basis of these results, we considered that the difference in the electron density of Co^(0)^ species, namely, electron-enrich Co by Ge via alloying, is the key factor for the remarkable enhancement in the catalytic activity.

The reaction mechanism of AB hydrolysis has been reported as follows: (1) the formation of an activated complex between AB and the metal surface, (2) B–N bond scission assisted by H_2_O attack, and (3) hydrolysis of the resulting BH_3_ moiety to form H_2_ and ${\text{BO}}_{2}^{-}$ (Xu and Chandra, 2006; Mahyari and Shaabani, 2014). It is also known that H atoms bound to the B and N atoms are slightly electropositive and electronegative, respectively, due to the different electronegativities of B and N. Therefore, it is likely that the alloy surface with polar active sites (Co^δ−^–Ge^δ+^) facilitates the formation of the active complex (1), thus enhancing the following steps and the overall reaction rate. A similar reaction mechanism has also been reported in the system of Ru^δ−^–Ni^δ+^ bimetallic catalysts for AB hydrolysis (Mori et al., 2016).

## Conclusion

In this study, we prepared a series of Ni- and Co-based bimetallic catalysts (Ni–M/SiO_2_ and Co–M/SiO_2_; M = Ga, Ge, Sn, and Zn) and tested them in H_2_ production from AB in water or methanol. Catalytic activity for hydrogen production is enhanced by the addition of second metals except Sn. Particularly, the addition of Ge to Co enables great enhancement in the catalytic activity, namely a five-fold higher *r*_H2_ than the monometallic Co/SiO_2_ catalyst. The active species is electron-enriched Co atoms constituting the Co_0.8_Ge_0.2_ solid solution alloy phase. The Ni–Zn/SiO_2_ catalyst exhibited an *r*_cat[AB]_ value higher than those ever reported for hydrolysis of AB to the best of our knowledge. The Ni_0.75_Zn_0.25_ solid solution alloy phase acts as the active species.

## Author Contributions

SF designed the research and experiments. GN performed all the experiments. TT managed the HAADF-STEM-EDS analysis. SF and TK prepared the manuscript.

### Conflict of Interest Statement

The authors declare that the research was conducted in the absence of any commercial or financial relationships that could be construed as a potential conflict of interest.
